# Comparison of Proteomics Profiles Between Xenografts Derived from Cell Lines and Primary Tumors of Thyroid Carcinoma

**DOI:** 10.7150/jca.50897

**Published:** 2021-01-31

**Authors:** Luo Fang, Yu-jia Liu, Yi-wen Zhang, Zong-fu Pan, Li-ke Zhong, Lie-hao Jiang, Jia-feng Wang, Xiao-wei Zheng, Ling-ya Chen, Ping Huang, Ming-hua Ge, Zhuo Tan

**Affiliations:** 1 Department of Head and neck & thyroid surgery, Zhejiang Provincial People's Hospital; People's Hospital of Hangzhou Medical College, 310010, Hangzhou, Zhejiang, China.; 2 Department of Pharmacy, The Cancer Hospital of the University of Chinese Academy of Sciences (Zhejiang Cancer Hospital), Institute of Basic Medicine and Cancer (IBMC), Chinese Academy of Sciences, 1# Bashan East Road, 310022, Hangzhou, China.; 3 Department of Pharmacy, Zhejiang Provincial People's Hospital; People's Hospital of Hangzhou Medical College, 310010, Hangzhou, Zhejiang, China.; 4 Key Laboratory of Endocrine Gland Diseases of Zhejiang Province, 310010, Hangzhou, Zhejiang, China.

**Keywords:** Proteomics, Cell-line Derived Xenografts, Primary Tumor, Thyroid Cancer.

## Abstract

Patient-consistent xenograft model is a challenge for all cancers but particularly for thyroid cancer, which shows some of the greatest genetic divergence between human tumors and cell lines. In this study, proteomic profiles of tumor tissues from patients, included anaplastic thyroid carcinoma (ATC) and papillary thyroid carcinoma, and xenografts (8305C, 8505C, FRO, BAPAP and IHH4) were obtained using HPLC-tandem mass spectrometry and compared based on all proteins detected (3,961), cancer-related proteins and druggable proteins using pairwise Pearson's correlation analysis. The human tissue showed low proteomic similarity to the ATC cell lines (8305C, r = 0.344-0.416; 8505C, 0.47-0.579; FRO, 0.267-0.307) and to PTC cell lines (BCPAP, 0.303-0.468; IHH4, 0.262-0.509). Human tissue showed the following similarity to cell lines at the level of 135 cancer-related pathways. The ATC cell lines contained 47.4% of the cancer-related pathways (19.26%-33.33%), while the PTC cell lines contained 40% (BCPAP, 25.93%; IHH4, 28.89%). In patient tumor tissues, 44-60 of 76 and 52-53 of 93 druggable proteins were identified in ATC and PTC tumors, respectively. Ten and 29 druggable proteins were not identified in any of the ATC and PTC xenografts, respectively. We provide a reference for CDX selecting in* in vivo* studies of thyroid cancer.

## Introduction

The most commonly used preclinical *in vivo* model of cancer is a cell line-derived xenograft (CDX), which is generated by transplanting human tumor cells into immunocompromised mice. While CDX studies have generated valuable insights, CDX tissue and tumor patients differ in their behavior, making CDX inadequate for understanding and predicting human tumor behavior and response to therapy 1. CDX tissue differs genetically from patient tissue because CDX tissue is derived from a single patient, who differs genetically from other patients with the same cancer, and because cell lines is vulnerable to selected evolution 2, 3 and genomic instability 4. CDX and original human tumor tissue differ not only in their intrinsic genetics from cell line but also in their mechanisms of growth. For example, a proteomic study found that six of 10 pathways differed substantially between high-grade glioma cell lines and their CDX's 5.

Selecting appropriate cell lines is essential for accurate cancer studies, but choosing the best cells is often purely empirical. Common cell lines are often used, but their genetic consistency with tumor samples is often unclear. Ovarian cancer, for example, may be better studied using some rarely used cell lines than commonly used lines 6. Systematic analysis of the similarities and differences between CDX's and human tumors may help guide the selection of appropriate CDX's to mimic tumors for basic cancer research and translational studies.

Towards this goal, various cancer cell lines have been evaluated, as have pooled analysis of various cancers 1, 6-12 or specific types of solid tumors, including breast cancer, hepatocellular carcinoma, and melanoma 13-15. Moderate correlation was observed between mRNA levels in cell lines and primary solid tumors, with a median correlation coefficient of 0.6 12. But, the lowest corresponding coefficient (only 0.29) was found in thyroid cancer and <0.3 for the two subtypes of thyroid cancer, papillary thyroid carcinoma (PTC) and anaplastic thyroid carcinoma (ATC) 16. PTC is more common and involves well-differentiated tumors and good prognosis, while ATC is less common and involves less differentiated, more aggressive tumors. Therefore, the proteomic consistence was also need to be clear.

To provide a rational basis for selecting the most appropriate CDX for thyroid cancer studies, we performed proteomics profiling of ATC and PTC tumors as well as several CDX's (ATC: 8505C, 8305C, FRO; PTC: BCPAP, IHH4). The two types of cancer tissue were compared in terms of total proteins, cancer-related pathways and druggable proteins. The results lead us to recommend certain cell lines over others for *in vivo* studies of thyroid cancer.

## Materials and Methods

### Primary tumor tissues

Fresh frozen samples of primary tumor were obtained from five ATC patients (4 men, 1 woman) and 28 PTC patients (13 men, 15 women) who underwent surgery in Zhejiang Cancer Hospital in Hangzhou, China (Table 1). All specimens were flash-frozen after tissue type had been confirmed. This study was approved by the Ethics Committee of Zhejiang Cancer Hospital and performed according to the Declaration of Helsinki and Good Clinical Practice guidelines. Patients provided written informed consent for their tissue to be used in this study.

### Cell lines and xenografts

Five thyroid carcinoma cell lines were used in this study: the ATC cell lines 8305C, 8505C, and FRO were obtained from the Shanghai Cell Bank of the Chinese Academy of Sciences (Shanghai, China); while the PTC cell lines BCPAP and IHH4 were obtained from professor Haixia Guan (Department of Endocrinology and Metabolism, The First Affiliated Hospital of China Medical University, Shenyang, P.R. China). Cell lines 8305C and 8505C were cultured in DMEM, while the other lines were cultured in RPMI-1640. In all cases, the medium was supplemented with streptomycin (100 U/mL) and penicillin (100 U/mL) as well as fetal bovine serum (FBS) to a final concentration of 5% (FRO) or 10% (all other lines). Cultures were incubated at 37 °C in a humidified atmosphere of 5% CO_2_.

Female BALB/c nude mice weighing 16-22 g [Animal License No. SCXK-(Shanghai)-2015000521727] were obtained from the Shanghai Laboratory Animal Center (Shanghai, China) and housed at the Center for Drug Safety Evaluation at Zhejiang University [Hangzhou, China; Animal License No. SYXK (Zhejiang) 2012-0178]. Cultured cell lines were subcutaneously injected into the right flank. Once tumor volumes reached about 100 mm^3^, animals were sacrificed and tumor tissues were harvested and homogenized. Animal studies were conducted according to the National Research Council's guidelines and approved by the Committee on the Ethics of Animal Experiments of Zhejiang Cancer Hospital and the Center for Drug Safety Evaluation at Zhejiang University.

### Proteomic analysis

Total proteins from tumor tissues (25 mg) were extracted and digested with trypsin according to the Filter-Aided Sample Preparation method. The digest was desalted and analyzed on a liquid chromatography-mass spectrometry system consisting of a Nano-nLC 1000 system (Thermo Fisher Scientific) connected to a linear quadrupole ion trap-Orbitrap (LTQ Orbitrap Elite) mass spectrometer (Thermo Fisher Scientific), which was equipped with a Proxeon NanoSpray Flex Ion Source (Thermo Fisher Scientific). Peptides were eluted on an Acclaim PepMap RSLC column (15 cm, 50 μm, 2 μm) using a 150 min-last gradient elution program involving acetonitrile and 0.1% (v/v) formic acid at a flow rate of 250 nL/min. Optimized mass spectrometry conditions were as follows: full scan mass analysis; *m/z* range, 300-2,000; resolving power, 60,000 (at *m/z* 400, FWHM, 1-second acquisition); ion spray voltage, 1.8 kV; ion source temperature, 300 ℃; and collision energy, 35 eV. Tandem mass spectrometry was performed with rolling collision energy for the top 20 most intense precursor ions with charge states of at least 2+. Mass and composition of precursor and fragment ions were analyzed using Thermo Xcalibur 3.0 software (Thermo Fisher Scientific).

### Protein identification and quantification

Raw spectral data were processed and quantitative ratios determined using PEAKS Studio 8 (Bioinformatics Solutions); parent mass tolerance was 15.0 ppm and fragment mass tolerance was 0.8 Da. Results were compared against UNIPROT databases of *Piriformospora* and *Brassica* (updated to April 2017). Protein identifications were accepted if the false detection rate (FDR) was <1% and if the score -10×log_10_ P was >20. The minimum number of unique peptides was set to 1, and the maximum number of post-translational modifications was set to 3. ANOVA was used to assess differences for statistical significance.

### Determination of cancer-related pathways and druggable proteins

Cancer-related genes were determined by mining abstracts indexed in MEDLINE up to 18 September 2017 using the text-mining engine DiGseE 17. Critical pathways closely related to cancer were identified using DAVID Bioinformatics Resources 6.8. Statistical significance and significant enrichment were defined as *p* < 0.10. The list of 672 human proteins directly related to the mechanisms of action of drugs approved by the US Food and Drug Administration was obtained from Drugbank (Table S1).

### Similarity evaluation of primary and xenograft tumors

Human primary PTC or ATC tumors were compared with the corresponding CDX models in terms of their proteomic profiles or cancer-related pathways. This comparison was performed based on the pairwise Pearson correlation coefficient as described 12, here applied to the normalized expression levels of total detected proteins or proteins enriched in the particular cancer-related pathway under consideration. Similarity at the proteomics level was calculated between each patient and each CDX, while similarity at the level of individual cancer-related pathways was calculated between all ATC or PTC patients and each CDX. In brief, a linear model from the Linear Models for Microarray Data (LIMMA) package was fitted, and the average fold-difference for each protein between primary and xenograft tumors was calculated. Then, the pairwise Pearson's correlation coefficient between tumors and cell lines was calculated based on the fold-differences for all proteins or only for the proteins involved in a certain pathway. Similarity of cancer-related pathways between tumors and CDX's were assessed using the Mann-Whitney U test.

### Stratification of cell lines based on desirability for studies of cancer-related pathways

The cell lines were stratified into three classes based on their desirability as *in vivo* models for specific pathway research, as measured by the r-value: (1) not recommended, referring to cell lines showing only weak similarity (r < 0.25) for specific pathways; (2) recommended, referring to cell lines showing strong similarity (r > 0.60), or in the event that all cell lines fail to reach 0.60, the cell line with the highest r-value; (3) priority recommended, referring to the cell line with the highest r-value that is also >0.60.

## Results

A total of 3,961 proteins were identified from tumor samples.

### Proteomics-based similarity between primary and xenograft tumors

**Figure 1:**
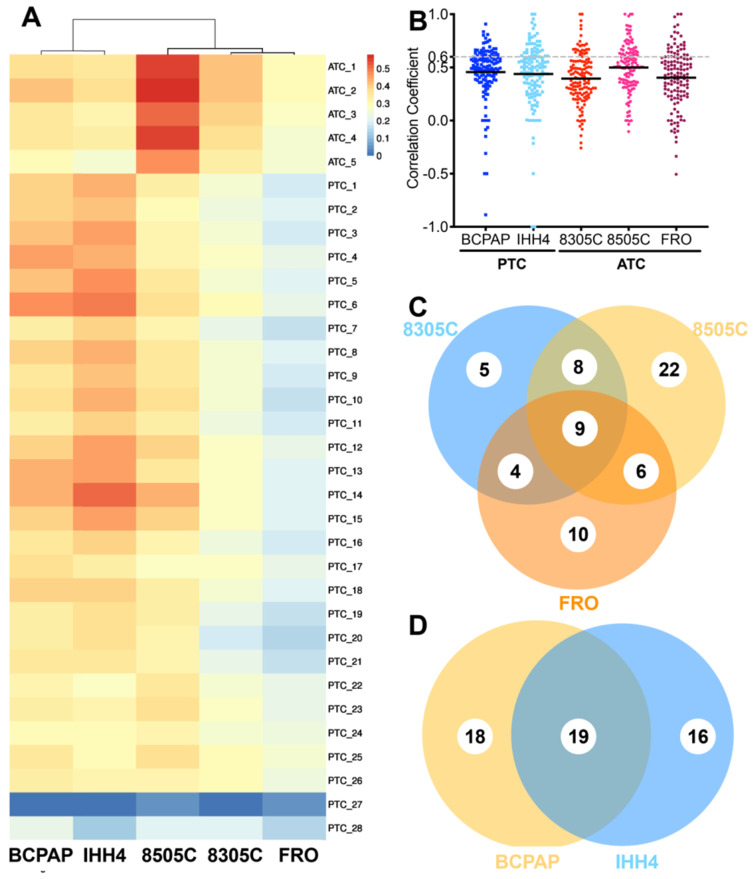
** Correlation of individual cancer-related pathways between cell line-derived xenografts and primary tumors of anaplastic thyroid carcinoma (ATC) or papillary thyroid carcinoma (PTC).** A. Comparison of proteomic profiles between four cell line-derived xenografts (columns) and primary tumors (rows). Heat map coloring is used to indicate the Pearson correlation coefficient. B. Scatter plot of correlation coefficients for individual cancer-related pathways between cell line-derived xenografts and primary tumors. C-D, Venn diagrams showing overlap of highly consistent pathways between primary and xenograft tumors in (C) PTC and (D) ATC.

After searching 16,167 publications, 4,149 cancer-related genes were identified, of which 2,350 genes were reported by at least two publications. Based on these 2,350 genes, 135 cancer-related pathways were identified (Table S2). The CDX's in this study showed weak correlations with primary tumors based on total of detectable proteins (Figure 1A): median correlation coefficients were 0.396 (range, 0.344-0.416) for 8305C, 0.278 (range, 0.267-0.307) for FRO, 0.366 (range, 0.303-0.468) for BCPAP, 0.397 (range, 0.262-0.509) for IHH4, and slightly higher for 8505C at 0.545 (range, 0.470-0.579).

Correlations were also weak based on proteins in cancer-related pathways (Figure 1B). Median correlation coefficients for 135 pathways were 0.43 (range, -0.16-1.00) in the case of ATC and 0.450 (range, -0.50-0.86) in the case of PTC. The 8505C cell line seemed more similar to ATC patient tissue (r = -0.11-1.00 and 0.36 for quartile 1, 0.51 for quartile 2, and 0.64 for quartile 3) than 8305C (r = -0.26-1.00, 0.26 for quartile 1, 0.39 for quartile 2, 0.56 for quartile 3, *p* < 0.0001) or FRO (r = -0.16-1.00, 0.32 for quartile 1, 0.43 for quartile 2, 0.55 for quartile 3, *p* = 0.0017). BCPAP (r = -0.89-0.91, 0.39 for quartile 1, 0.50 for quartile 2, 0.61 for quartile 3) seemed slightly more similar to PTC patient tissue than IHH4 (r = -1.0-1.0, 0.30 for quartile 1, 0.49 for quartile 2, 0.61 for quartile 3, *p* = 0.0607).

Fewer than one of every three pathways showed strong similarity (>0.60) between primary tumors and CDX's; the proportion was 19.26% for 8305C, 33.33% for 8505C, 22.96% for FRO, 25.93% for BCPAP, and 28.89% for IHH4. The two PTC cell lines recapitulated 54 cancer-related pathways (40.0%) with high consistency; the three ATC cell lines, 64 (47.4%). In contrast, the proportion of cancer-related pathways in xenograft tumors showing low consistency (<0.25) with primary tumors was 18.52% in 8305C, 31.11% in 8505C, 22.22% in FRO, 12.59% in BCPAP, and 27.41% in IHH4 (Figure 1C-D).

**Figure 2:**
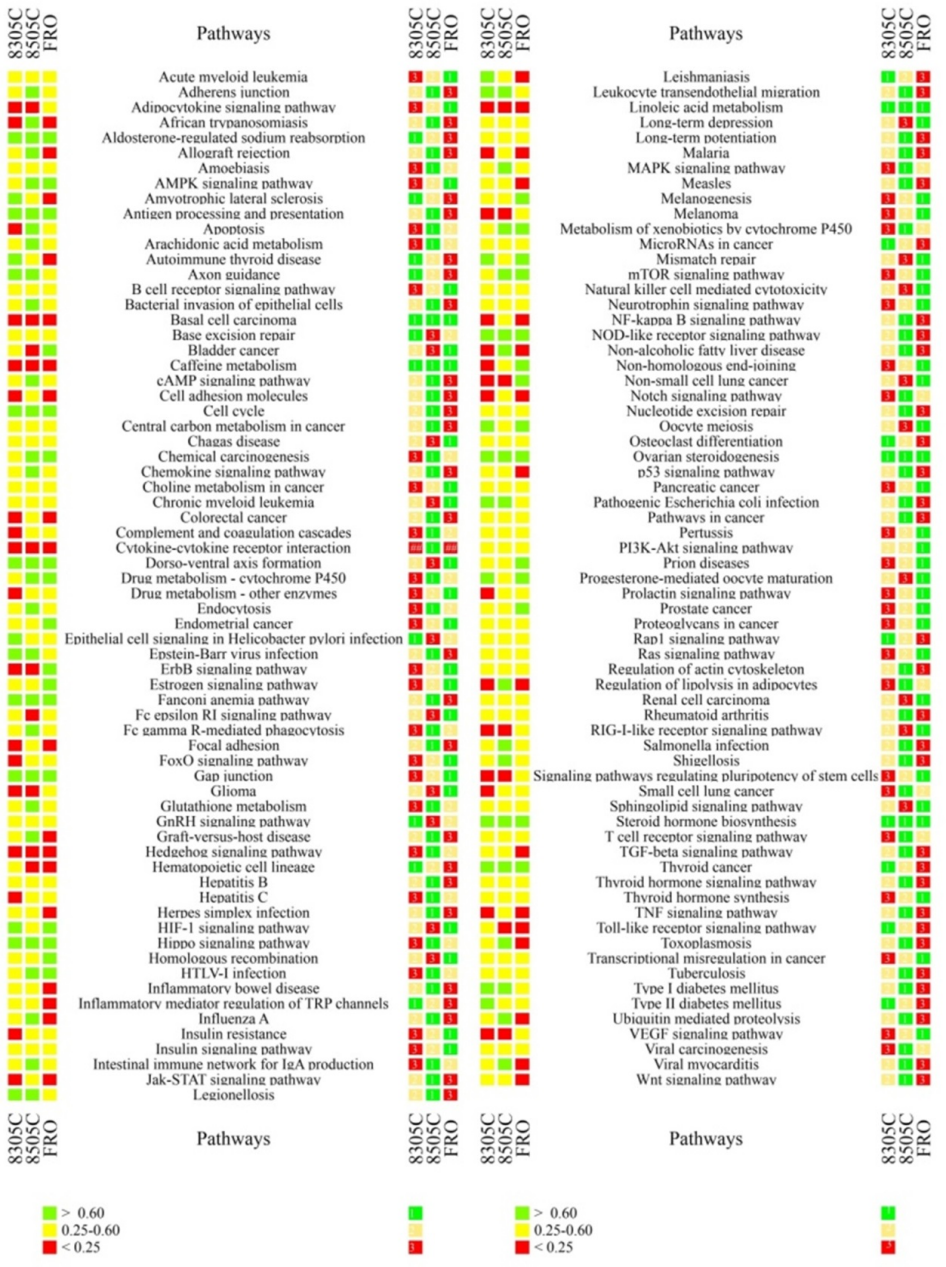
** Ranking of individual cancer-related pathways based on correlation coefficients between primary and xenograft ATC tumors.** The colored cubes on the left indicate the correlation coefficient as >0.60 (green), 0.25-0.60 (gray), or <0.25 (red). The colored cubes on the right indicate the consistency as high (green), moderate (yellow) or low (red).

In the case of ATC, many key cancer pathways showed poor consistency between primary tumors and at least one CDX (Figure 2). These pathways included those involving the RIL receptor, FoxO, VEGF, ErbB, Notch, Jak-STAT, TGF-beta, p53, Wnt, Hedgehog, and Toll-like receptor; as well as those regulating pluripotency of stem cells, apoptosis, focal adhesion, cell adhesion, thyroid disease, or ubiquitin-mediated proteolysis. Similarly, many key pathways in PTC showed poor consistency between primary tumors and at least one CDX. These pathways included those involving p53, microRNAs, thyroid hormone and Hedgehog; as well as those regulating pluripotency of stem cells, mismatch repair, base excision repair, nucleotide excision repair, glutathione metabolism, small cell lung cancer; and many immunological pathways involving Fc epsilon RI, hepatitis C, complement and coagulation cascades, NF-kappa B, TNF, and autoimmune responses. In addition, many pathways showing poor consistency between primary and xenograft tumors are known to be important in bladder cancer, melanoma, glioma, non-small cell lung cancer, small cell lung cancer, colorectal cancer, and basal cell carcinoma (Table 2,4).

**Figure 3:**
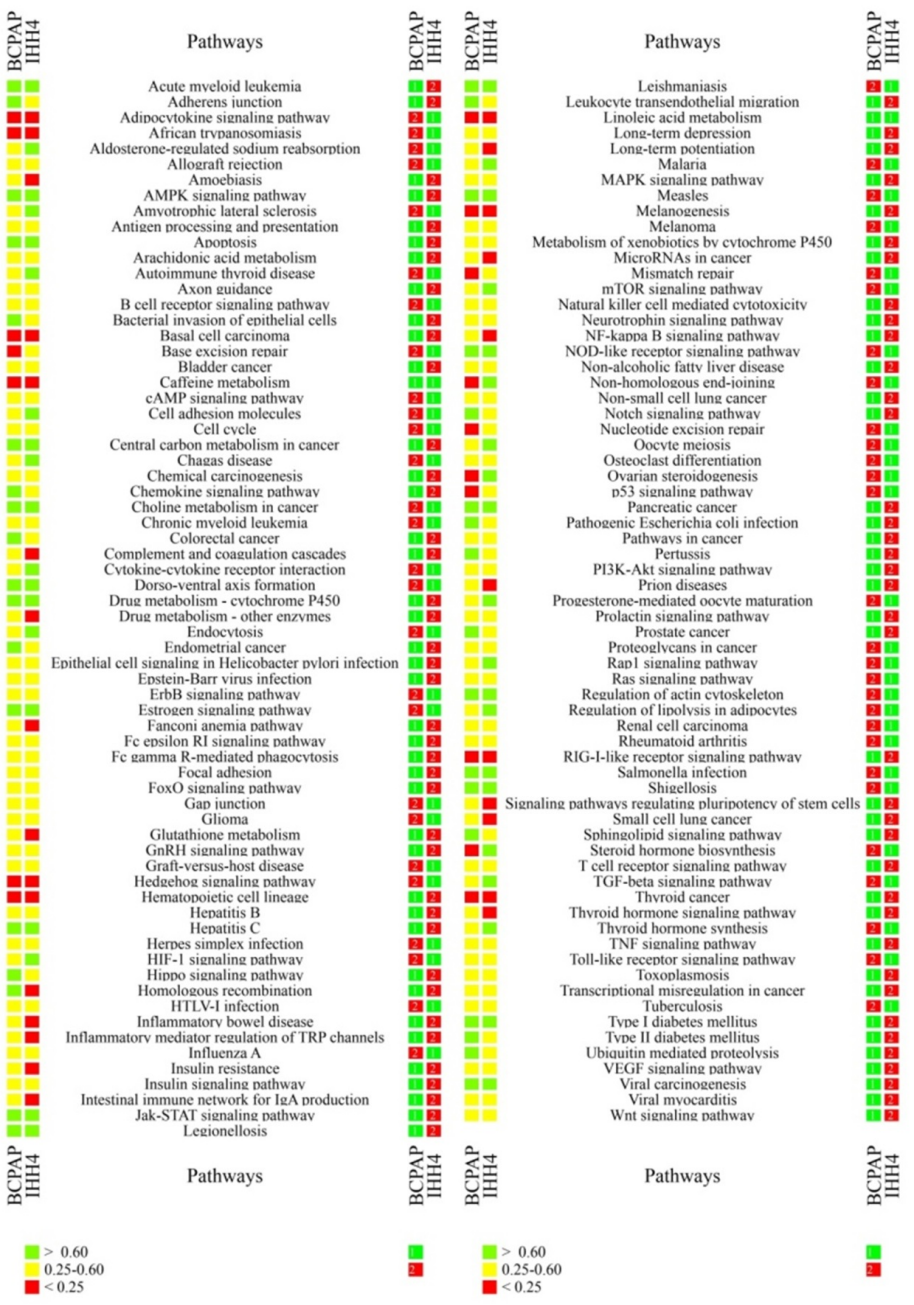
** Ranking of individual cancer-related pathways based on correlation coefficients between primary and xenograft PTC tumors.** The colored cubes on the left indicate the correlation coefficient as >0.60 (green), 0.25-0.60 (gray), or <0.25 (red). The colored cubes on the right indicate the consistency as high (green) or low (red).

Cell lines 8305C, 8505C, and FRO emerged as better models for studying 37, 79, or 61 pathways, respectively. These pathways included ones showing low and high consistency between primary and xenograft tumors, and they included several pathways that showed greater similarity in one cell line than in the other two: 11 in cell line 8305C, 34 in 8505C, and 32 in FRO (Table 3). Cell lines BCPAP and IHH4 emerged as better models for studying 89 and 67 pathways, although approximately half of them (54 in BCPAP, 67 in IHH4) did not show high consistency between primary and xenograft tumors (Table 3,5). The spectrum of consistency is shown in Figure 2 and Figure 3.

### Druggable proteins

**Figure 4:**
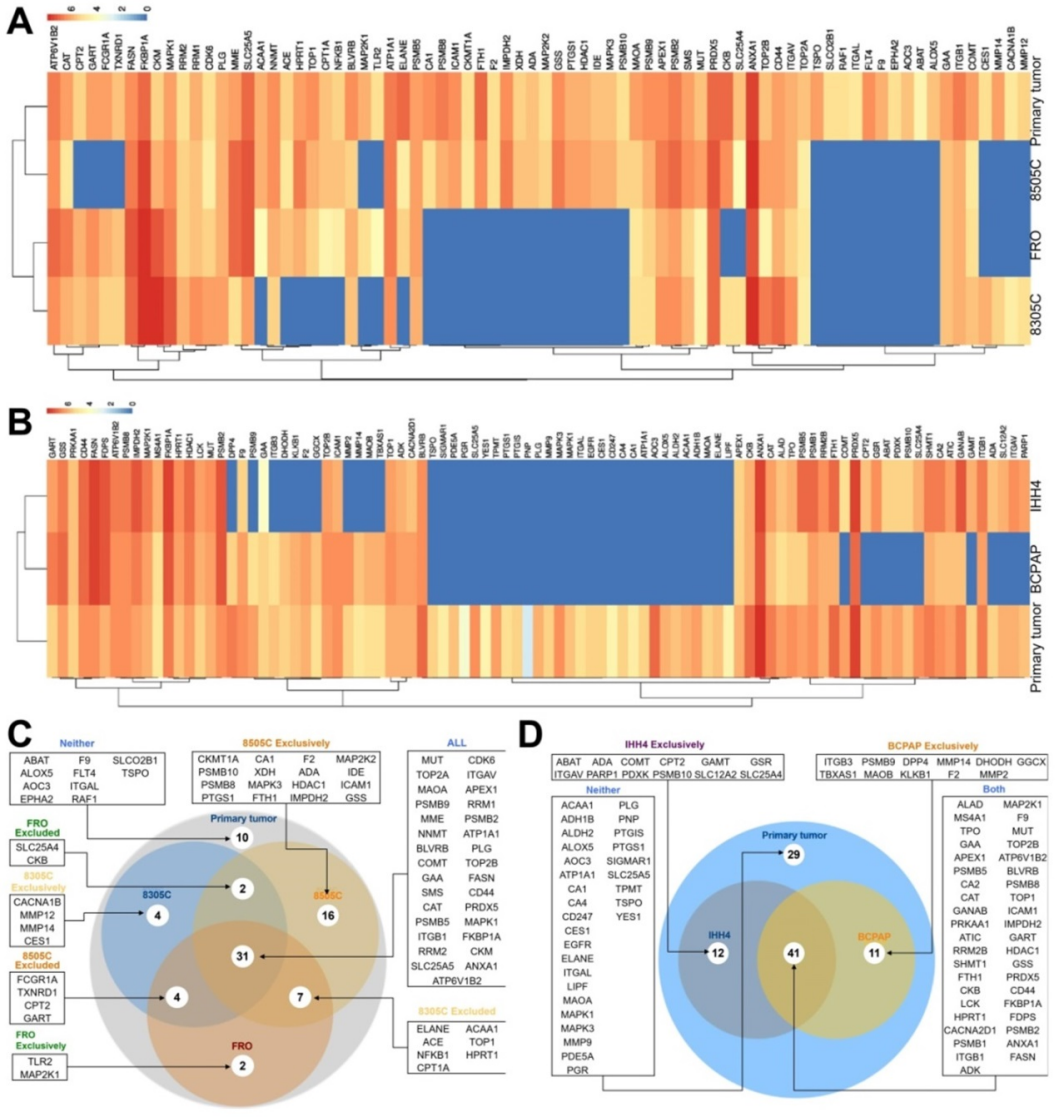
** Expression of druggable proteins in primary and xenograft tumors of thyroid cancer.** A-B, Heatmap of druggable protein levels detected in patient tumors and CDXs of (A) ATC and (B) PTC. C-D, Venn diagram showing overlap of druggable proteins detected in CDXs of (C) ATC and (D) PTC.

Of 672 druggable proteins, 76 were identified in primary ATC tumors and 93 in PTC tumors. Among the three ATC CDX's, 31 proteins were detected in all three, while 10 were not detected in any. A total of 60 proteins were identified in 8505C, 48 in FRO, and 44 in 8305C. Of the 93 druggable proteins detected in primary PTC tumors, 12 were also detected in IHH4, 11 in BCPAP, and 41 in both cell lines. A total of 29 proteins were not detected in either cell line (Figure 4).

## Discussion/Conclusion

The phenotypic discrepancies between preclinical models and patients is a challenge in basic cancer studies and in efforts to discover and develop antineoplastic agents 18-21. This has motivated searches for xenograft tumor models that show maximum similarity to primary human tumors. These efforts are limited by lack of understanding about molecular details of cancer cells 22, and they have been less successful with thyroid cancer. Therefore we employed proteomics and other analyses to compare total protein profiles as well as proteins involved in specific cancer-related pathways between primary thyroid cancer tumors and xenograft tumors derived from several cell lines. Proteomic profiles of 28 primary human PTC tumors and five ATC tumors were compared with proteomic profiles from two PTC CDX's (BCPAP, IHH4) and three ATC CDX's (8305C, 8505C, FRO). Similarity between the two kinds of tumor tissue was assessed according to total proteins, cancer-related proteins, and druggable proteins.

CDX's were not very similar to ATC primary tumors when the complete proteomic profiles were considered: the correlation coefficient was 0.39 for 8305C, 0.53 for 8505C, and 0.29 for FRO. Similar results were observed for PTC tumors, where the correlation coefficient was 0.35 for BCPAP and 0.37 for IHH4. CDX's other than 8505C showed low consistency, which agrees with studies comparing mRNA levels between human thyroid cancer cell lines and human thyroid tumors. Genomic profiles showed a correlation coefficient of only approximately 0.29 between thyroid cancer cell lines and human tumors, and this coefficient is the lowest among all types of cancer. Another study found a Spearman correlation coefficient <0.3 between ATC cell lines and primary tumors, and an even lower coefficient <0.1 between PTC lines and primary tumors 16.

Similarity between primary and xenograft tumors was further investigated at the level of individual cancer-related pathways. A total of 135 cancer-related pathways were identified based on KEGG analysis of gene enrichment in publications from MEDLINE. The similarity of each pathway was calculated based on the expression levels of all involved proteins more than differential proteins. The two PTC cell lines and three ATC cell lines in this study covered 54 (40.0%) and 64 (47.4%) of cancer-related pathways. Overall, these pathways showed moderate similarity between CDX's and human tumors. More than 70% of pathways showed low consistency between the two tumor types. Even in the cell line showing the highest consistency, 8505C, only 33.33% of pathways showed high consistency with primary tumors. Up to one quarter of pathways (11.9-24.4%) showed correlation coefficients <0.25. Priority recommended cell lines were identified for approximately 40 percent of 135 pathways (53 in PTC and 64 in ATC). BCPAP was identified as being not recommended for 15 (11.11%) of pathways with r < 0.25; IHH4, 26 (19.26%) pathways; 8305C, 14 (10.37%) pathways; 8505C for 32 (23.70%) pathways; and FRO, 33 (24.44%) pathways.

The heterogeneous consistency between primary and xenograft tumors at the level of individual pathways suggests that no single CDX model fits most pathways important in thyroid cancer. Our finding that many immunology-related pathways, such as those involving Toll-like receptors, TNF, or NF-κB, show low consistency between primary and xenograft tumor types likely reflects the immunosuppression intrinsic to nude mice. The lack of consistency in the hedgehog pathway between primary and xenograft PTC or ATC tumors is an important reminder of the need to choose cancer cell lines cautiously. The hedgehog pathway is activated to stimulate the growth and spread of many types of solid tumors, including thyroid cancer. It may also affect patient prognosis, but this remains controversial 23, 24. Inhibiting hedgehog signaling by knocking down key genes or applying small-molecule inhibitors significantly reduces cell proliferation 24. In fact, two small-molecule inhibitors of hedgehog signaling, cyclopamine and GDC-0449, have been explored *in vitro* as candidates for thyroid cancer treatment 25, 26. Interestingly, the pathway “thyroid cancer” showed low similar between primary PTC tumors and IHH4 or BCPAP-derived xenografts.

Although few drugs have been approved for treating thyroid cancer, we compared the profile of 672 FDA-approved druggable proteins between primary and xenograft thyroid tumors. These proteins target enzymes, transporters, voltage-gated ion channels, G-protein coupled receptors, nuclear receptors and CD markers. A total of 93 druggable proteins were detected in primary PTC tumors, and only 44% of druggable proteins were expressed in both PTC cell lines. More than 30% of proteins were not detected in either of these lines. A total of 76 druggable proteins were detected in primary ATC tumors, of which 31 were detected in all three ATC cell lines, 22 were detected in at least one line, and 13 were detected in two lines. On the other hand, 10 proteins (ABAT, ALOX5, AOC3, EPHA2, F9, FLT4, ITGAL, RAF1, SLCO2B1, TSPO) were not detected in any of the three lines. The differential expression-level of targeted proteins might partly indicated different response to approved drugs among xenograft models and primary tumors.

The proteomic consistency between human thyroid cancer tissue and xenografts derived from human thyroid cancer cell lines was investigated based on total protein, cancer-related proteins, and druggable proteins. The results indicate rather low consistency between primary and xenograft tumors. Most CDX's showed poor correlation with primary tumors (r < 0.4), while the 8505C-derived xenograft showed medium correlation (0.545). At the level of individual cancer-related pathways, fewer than one third of pathways were highly similar between primary and xenograft tumors (>0.60); 12.6-31.1% of pathways showed low similarity (<0.25). Differences in druggable proteins were also observed between primary and xenograft tumors, as well as among xenograft tumors.

Key genes related to thyroid carcinoma, such as RET, p53, RAS, BRAF and β-catenin, should be additionally addressed. Therefore, the responding signaling pathway, included p53 signaling pathway, RAS signaling pathway, MAPK signaling pathways, WNT signaling pathway, and PI3K-AKT/RAS signaling pathway were additionally discussed. Most signaling pathways were with moderate correlation (r=0.40-0.53) between xenografts and patients, except poor consistancy of p53 in PTC (r=0.23).

There're also limitations of subcutaneous xenograft tumor model. For example, subcutaneous tumor size variability was generally very low, and the proliferation rate was slower than orthotopic tumor model. These differences in overall growth indicated the crucial role of an appropriate microenvironment and organ-specific angiogenesis in facilitating initial tumor growth and perhaps later aggressiveness. Another important reason is that subcutaneous xenograft tumor model do not clearly reproduce the primary site of cancers and nor do they represent the common sites of metastasis. On the contrary, orthotopic tumor placement appears to better mimic the microenvironment, morphology, growth, and metastatic patterns of human cancer. Therefore, these limitations may also be important reasons for the poor correlation between the CDX model and the primary model in thyroid tumor in our study. Moreover, the mutation status, express levels of key genes should be focus in future studies.

## Supplementary Material

Supplementary figures and tables.

## Figures and Tables

**Table 1:** Clinical and pathological characteristics of patients who donated primary tumor tissue

Patient NO.	Histology	Sex	Age (years)	Tumor size (cm)
1	ATC	M	75	5.2
2	ATC	M	54	6.0
3	ATC	M	47	2.5
4	ATC	F	72	7.0
5	ATC	M	77	10.0
6	PTC	M	65	1.0
7	PTC	F	68	2.0
8	PTC	M	58	1.6
9	PTC	M	54	1.4
10	PTC	M	54	1.5
11	PTC	F	67	0.6
12	PTC	F	43	4.5
13	PTC	M	42	2.4
14	PTC	M	58	4.0
15	PTC	F	58	5.0
16	PTC	M	58	2.3
17	PTC	F	59	2.5
18	PTC	F	22	2.5
19	PTC	M	66	3.0
20	PTC	F	21	2.3
21	PTC	F	25	4.5
22	PTC	F	47	1.2
23	PTC	F	28	2.5
24	PTC	M	52	3.5
25	PTC	F	45	0.6
26	PTC	F	62	2.0
27	PTC	F	56	1.7
28	PTC	F	50	0.9
29	PTC	F	54	0.9
30	PTC	M	48	0.6
31	PTC	M	48	0.8
32	PTC	M	55	1.0
33	PTC	M	53	0.8

PTC, papillary thyroid cancer; ATC, anaplastic thyroid cancer; F, female; M, male.

**Table 2:** Cancer-related pathways grouped based on their similarity between primary and xenograft ATC tumors

	Correlation coefficient						
	>0.6			0.25-0.6(n=35)	<0.25		
	8505C	8305C	FRO	Any cell lines	8505C	8305C	FRO
8505C	Non-alcoholic fatty liver disease, cAMP, Toxoplasmosis, MAPK, Fc gamma R-mediated phagocytosis, Glutathione metabolism, Intestinal immune network for IgA production, Endocytosis, Chemokine, Influenza A, Viral myocarditis, Allograft rejection, Graft-versus-host disease, Adherens junction, Regulation of lipolysis in adipocytes, Shigellosis, Bacterial invasion of epithelial cells, Arachidonic acid metabolism, Ubiquitin-mediated proteolysis, *Salmonella* infection, African trypanosomiasis, Apoptosis	Leukocyte transendothelial migration, Axon guidance, Epstein-Barr virus infection, Pathogenic *Escherichia coli* infection, Type I diabetes mellitus, Legionellosis	AMPK, mTOR, HTLV-I infection, Metabolism of xenobiotics by cytochrome P450, Chemical carcinogenesis, Drug metabolism - cytochrome P450	Long-term depression, Sphingolipid, GnRH, Pertussis, Base excision repair, Thyroid hormone synthesis, Pancreatic cancer, Acute myeloid leukemia, Chagas disease, Chronic myeloid leukemia, Insulin, PI3K-Akt, Natural killer cell-mediated cytotoxicity, Amoebiasis, Choline metabolism in cancer, Proteoglycans in cancer, MicroRNAs in cancer, Renal cell carcinoma, B cell receptor, Long-term potentiation, Transcriptional misregulation in cancer, Hepatitis B, Thyroid hormone, Tuberculosis, Viral carcinogenesis, Ras, Pathways in cancer, Neurotrophin, Central carbon metabolism in cancer, Rap1, Osteoclast differentiation, Nucleotide excision repair, T cell receptor, Regulation of actin cytoskeleton, Rheumatoid arthritis	Fc epsilon RI, Bladder cancer	Melanoma, Adipocytokine, Glioma, RIG-I-like receptor, Signaling pathways regulating pluripotency of stem cells, VEGF, Non-small cell lung cancer, ErbB	Toll-like receptor, Hematopoietic cell lineage
8305C	-	Amyotrophic lateral sclerosis, Epithelial cell signaling in *Helicobacter pylori* infection, Type II diabetes mellitus, Autoimmune thyroid disease, Leishmaniasis	HIF-1, Progesterone-mediated oocyte maturation, Mismatch repair, Oocyte meiosis		-	FoxO, Prolactin, Hepatitis C, Small cell lung cancer, Drug metabolism - other enzymes, Complement and coagulation cascades, Insulin resistance, Non-homologous end joining, Apoptosis	Focal adhesion, NF-kappa B, Notch, Colorectal cancer, TNF, Jak-STAT, Malaria, Cell adhesion molecules, Non-alcoholic fatty liver disease, Regulation of lipolysis in adipocytes, African trypanosomiasis
FRO	-	-	Bladder cancer, Non-small cell lung cancer, ErbB, Melanogenesis, Non-homologous end joining, Homologous recombination, Endometrial cancer, Prion diseases, Estrogen, Prostate cancer		-	-	TGF-beta, Inflammatory mediator regulation of TRP channels, Inflammatory bowel disease, Herpes simplex infection, p53, Wnt, Measles, Amyotrophic lateral sclerosis, Autoimmune thyroid disease, Leishmaniasis, Toxoplasmosis, Influenza A, Viral myocarditis, Allograft rejection, Graft-versus-host disease, Ubiquitin-mediated proteolysis
ALL	Thyroid cancer, Dorso-ventral axis formation, Gap junction, Antigen processing and presentation, Hippo, Cell cycle, Aldosterone-regulated sodium reabsorption, NOD-like receptor, Fanconi anemia pathway, Steroid hormone biosynthesis, Ovarian steroidogenesis				Hedgehog, Basal cell carcinoma, Caffeine metabolism, Linoleic acid metabolism		

**Table 3:** ATC-related pathways recommended for cell line-based studies based on consistency with CDX's

Cell line	Top 1 consistency			Non-top 1
	Low consistency	Moderate consistency	High consistency	
8505C	Hedgehog signaling pathway, Basal cell carcinoma, Caffeine metabolism, Linoleic acid metabolism	Inflammatory bowel disease, NF-kappa B signaling pathway, Jak-STAT signaling pathway, Malaria, Small cell lung cancer, Colorectal cancer, Focal adhesion, Notch signaling pathway, TGF-beta signaling pathway, TNF signaling pathway, Hepatitis C, Measles, p53 signaling pathfway, Amoebiasis, Herpes simplex infection, Cell adhesion molecules, Complement and coagulation cascades, Thyroid hormone signaling pathway, Long-term potentiation, Viral carcinogenesis, Hepatitis B, Tuberculosis, Wnt signaling pathway, T cell receptor signaling pathway, Nucleotide excision repair, Pathways in cancer, Central carbon metabolism in cancer, Regulation of actin cytoskeleton, Rheumatoid arthritis	African trypanosomiasis, Regulation of lipolysis in adipocytes, Non-alcoholic fatty liver disease, Allograft rejection, Graft-versus-host disease, Viral myocarditis, Apoptosis, Intestinal immune network for IgA production, Toxoplasmosis, Ubiquitin-mediated proteolysis, Glutathione metabolism, Influenza A, cAMP signaling pathway, Endocytosis, Chemokine signaling pathway, Leukocyte transendothelial migration, MAPK signaling pathway, Fc gamma R-mediated phagocytosis, Adherens junction, HTLV-I infection, Bacterial invasion of epithelial cells, Shigellosis, Epstein-Barr virus infection, Arachidonic acid metabolism, *Salmonella* infection, Type I diabetes mellitus, Legionellosis, Pathogenic *Escherichia coli* infection, Antigen processing and presentation, Metabolism of xenobiotics by cytochrome P450, Cell cycle, Chemical carcinogenesis, Hippo signaling pathway, Drug metabolism - cytochrome P450, Fanconi anemia pathway, NOD-like receptor signaling pathway, Ovarian steroidogenesis, Steroid hormone biosynthesis	AMPK signaling pathway, mTOR signaling pathway, Thyroid cancer, Axon guidance, Gap junction, Aldosterone-regulated sodium reabsorption, Dorso-ventral axis formation
8305C	Basal cell carcinoma, Caffeine metabolism, Linoleic acid metabolism	Hematopoietic cell lineage, Toll-like receptor signaling pathway, Inflammatory mediator regulation of TRP channels, GnRH signaling pathway, MicroRNAs in cancer, Base excision repair, Osteoclast differentiation, Rap1 signaling pathway	Amyotrophic lateral sclerosis, Autoimmune thyroid disease, Type II diabetes mellitus, Leishmaniasis, Epithelial cell signaling in *Helicobacter pylori* infection, Thyroid cancer, Axon guidance, Aldosterone-regulated sodium reabsorption, Ovarian steroidogenesis, Steroid hormone biosynthesis	Leukocyte transendothelial migration, Epstein-Barr virus infection, Mismatch repair, Type I diabetes mellitus, Legionellosis, Pathogenic *Escherichia coli* infection, HIF-1 signaling pathway, Oocyte meiosis, Gap junction, Antigen processing and presentation, Progesterone-mediated oocyte maturation, Cell cycle, Hippo signaling pathway, Fanconi anemia pathway, Dorso-ventral axis formation, NOD-like receptor signaling pathway
FRO	Basal cell carcinoma, Caffeine metabolism, Linoleic acid metabolism	Adipocytokine signaling pathway, Melanoma, Signaling pathways regulating pluripotency of stem cells, Fc epsilon RI signaling pathway, VEGF signaling pathway, RIG-I-like receptor signaling pathway, Glioma, FoxO signaling pathway, Pancreatic cancer, Prolactin signaling pathway, Pertussis, Drug metabolism - other enzymes, Sphingolipid signaling pathway, Proteoglycans in cancer, Insulin signaling pathway, Thyroid hormone synthesis, Long-term depression, PI3K-Akt signaling pathway, B cell receptor signaling pathway, Acute myeloid leukemia, Transcriptional misregulation in cancer, Chronic myeloid leukemia, Chagas disease, Choline metabolism in cancer, Insulin resistance, Natural killer cell mediated cytotoxicity, Ras signaling pathway, Renal cell carcinoma, Neurotrophin signaling pathway	Non-small cell lung cancer, Bladder cancer, ErbB signaling pathway, Non-homologous end joining, Melanogenesis, Endometrial cancer, Prion diseases, AMPK signaling pathway, mTOR signaling pathway, Mismatch repair, Estrogen signaling pathway, Homologous recombination, Prostate cancer, HIF-1 signaling pathway, Oocyte meiosis, Gap junction, Progesterone-mediated oocyte maturation, Dorso-ventral axis formation, Ovarian steroidogenesis, Steroid hormone biosynthesis	HTLV-I infection, Thyroid cancer, Metabolism of xenobiotics by cytochrome P450, Cell cycle, Chemical carcinogenesis, Hippo signaling pathway, Drug metabolism - cytochrome P450, Aldosterone-regulated sodium reabsorption, NOD-like receptor signaling pathway

**Table 4:** Cancer-related pathways grouped based on their similarity between primary and xenograft PTC tumors

	Correlation coefficient				
	>0.6		0.25-0.6	<0.25	
BCPAP	Sphingolipid, Ubiquitin-mediated proteolysis, Type II diabetes mellitus, Prostate cancer, Pertussis, Colorectal cancer, Endometrial cancer, Notch, Hippo, Leukocyte transendothelial migration, Bacterial invasion of epithelial cells, Homologous recombination, Adherens junction, Chemokine, Pathogenic Escherichia coli infection	Shigellosis, Regulation of actin cytoskeleton, Leishmaniasis, Viral carcinogenesis, Acute myeloid leukemia, Choline metabolism in cancer, Estrogen, Hepatitis C, NOD-like receptor, Measles, AMPK, Type I diabetes mellitus, Drug metabolism - cytochrome P450, Dorso-ventral axis formation, Pancreatic cancer, Legionellosis, Salmonella infection, Central carbon metabolism in cancer, Jak-STAT, Apoptosis	ErbB, HTLV-I infection, Glioma, Neurotrophin, TNF, Bladder cancer, Allograft rejection, Graft-versus-host disease, Osteoclast differentiation, Focal adhesion, Cell cycle, VEGF, Hepatitis B, Arachidonic acid metabolism, Toxoplasmosis, cAMP, B cell receptor, Chemical carcinogenesis, Non-small cell lung cancer, Herpes simplex infection, Proteoglycans in cancer, Gap junction, Influenza A, Epithelial cell signaling, Helicobacter pylori infection, Antigen processing and presentation, Fc gamma R-mediated phagocytosis, Ras, Viral myocarditis, Renal cell carcinoma, GnRH, Rheumatoid arthritis, Wnt, Pathways in cancer, Toll-like receptor, Melanoma, Insulin, PI3K-Akt, Non-alcoholic fatty liver disease, Epstein-Barr virus infection, Natural killer cell-mediated cytotoxicity, Tuberculosis, T cell receptor, Fc epsilon RI, MAPK, Chronic myeloid leukemia, Long-term depression, Prolactin, Metabolism of xenobiotics by cytochrome P450, Transcriptional misregulation in cancer, FoxO, Axon guidance, TGF-beta, Aldosterone-regulated sodium reabsorption, Cytokine-cytokine receptor interaction, Malaria, Progesterone-mediated oocyte maturation, mTOR, Amyotrophic lateral sclerosis, Cell adhesion molecules, Thyroid hormone synthesis, Endocytosis, Oocyte meiosis, HIF-1-, Chagas disease, Rap1, Regulation of lipolysis in adipocytes, Autoimmune thyroid disease	Mismatch repair, Base excision repair, Nucleotide excision repair, p53, Steroid hormone biosynthesis, Non-homologous end-joining, Ovarian steroidogenesis	Hedgehog, Thyroid cancer, Basal cell carcinoma, Linoleic acid metabolism, Caffeine metabolism, Hematopoietic cell lineage, African trypanosomiasis, Adipocytokine, RIG-I-like receptor, Melanogenesis
IHH4	Steroid hormone biosynthesis, Non-homologous end-joining, Ovarian steroidogenesis, TGF-beta, Aldosterone-regulated sodium reabsorption, Cytokine-cytokine receptor interaction, Malaria, Progesterone-mediated oocyte maturation, mTOR, Amyotrophic lateral sclerosis, Cell adhesion molecules, Thyroid hormone synthesis, Endocytosis, Oocyte meiosis, HIF-1, Chagas disease, Rap1, Regulation of lipolysis in adipocytes, Autoimmune thyroid disease			Glutathione metabolism, Long-term potentiation, Inflammatory mediator regulation of TRP, channels, Small cell lung cancer, Thyroid hormone, MicroRNAs in cancer, Signaling pathways regulating pluripotency of stem cells, Intestinal immune network for IgA production, Amoebiasis, Complement and coagulation cascades, Fanconi anemia pathway, Inflammatory bowel disease, NF-kappa B, Insulin resistance, Prion diseases, Drug metabolism - other enzymes	

**Table 5:** PTC-related pathways recommended for cell line-based studies based on consistency with CDX's

Cell line	Top 1 consistency			Non-top 1
	Low consistency	Moderate consistency	High consistency	
BCPAP	Thyroid cancer, Basal cell carcinoma, Caffeine metabolism, Linoleic acid metabolism, Hematopoietic cell lineage, RIG-I-like receptor signaling pathway, Melanogenesis	Glutathione metabolism, Long-term potentiation, Inflammatory mediator regulation of TRP channels, Neurotrophin signaling pathway, TNF signaling pathway, Small cell lung cancer, Thyroid hormone signaling pathway, Bladder cancer, MicroRNAs in cancer, Signaling pathways regulating pluripotency of stem cells, Intestinal immune network for IgA production, Focal adhesion, Amoebiasis, VEGF signaling pathway, Hepatitis B, Arachidonic acid metabolism, Toxoplasmosis, Chemical carcinogenesis, Non-small cell lung cancer, Complement and coagulation cascades, Epithelial cell signaling in *Helicobacter pylori* infection, Antigen processing and presentation, Fc gamma R-mediated phagocytosis, Viral myocarditis, GnRH signaling pathway, Fanconi anemia pathway, Wnt signaling pathway, Pathways in cancer, Insulin signaling pathway, PI3K-Akt signaling pathway, Inflammatory bowel disease, Non-alcoholic fatty liver disease, NF-kappa B signaling pathway, Insulin resistance, Epstein-Barr virus infection, Natural killer cell-mediated cytotoxicity, Prion diseases, T cell receptor signaling pathway, Fc epsilon RI signaling pathway, MAPK signaling pathway, Long-term depression, Prolactin signaling pathway, Metabolism of xenobiotics by cytochrome P450, Drug metabolism - other enzymes, Transcriptional misregulation in cancer, FoxO signaling pathway, Axon guidance	Sphingolipid signaling pathway, Ubiquitin-mediated proteolysis, Type II diabetes mellitus, Prostate cancer, Pertussis, Colorectal cancer, Viral carcinogenesis, Acute myeloid leukemia, Endometrial cancer, Notch signaling pathway, Hippo signaling pathway, Hepatitis C, Leukocyte transendothelial migration, Bacterial invasion of epithelial cells, Homologous recombination, AMPK signaling pathway, Adherens junction, Type I diabetes mellitus, Chemokine signaling pathway, Drug metabolism - cytochrome P450, Pancreatic cancer, Legionellosis, Central carbon metabolism in cancer, Pathogenic *Escherichia coli* infection, Jak-STAT signaling pathway, Apoptosis	Shigellosis, Regulation of actin cytoskeleton, Leishmaniasis, Choline metabolism in cancer, Estrogen signaling pathway, NOD-like receptor signaling pathway, Measles, Dorso-ventral axis formation, *Salmonella* infection
IHH4	Hedgehog signaling pathway, Caffeine metabolism, Linoleic acid metabolism, African trypanosomiasis, Adipocytokine signaling pathway	Mismatch repair, Base excision repair, Nucleotide excision repair, p53 signaling pathway, Non-homologous end joining, ErbB signaling pathway, HTLV-I infection, Glioma, Allograft rejection, Graft-versus-host disease, Osteoclast differentiation, Cell cycle, cAMP signaling pathway, B cell receptor signaling pathway, Herpes simplex infection, Proteoglycans in cancer, Gap junction, Influenza A, Ras signaling pathway, Renal cell carcinoma, Rheumatoid arthritis, Toll-like receptor signaling pathway, Melanoma, Tuberculosis, Chronic myeloid leukemia	Steroid hormone biosynthesis, Ovarian steroidogenesis, TGF-beta signaling pathway, Aldosterone-regulated sodium reabsorption, Cytokine-cytokine receptor interaction, Malaria, Progesterone-mediated oocyte maturation, mTOR signaling pathway, Amyotrophic lateral sclerosis, Cell adhesion molecules, Thyroid hormone synthesis, Endocytosis, Oocyte meiosis, HIF-1 signaling pathway, Chagas disease, Rap1 signaling pathway, Regulation of lipolysis in adipocytes, Autoimmune thyroid disease, Shigellosis, Regulation of actin cytoskeleton, Leishmaniasis, Choline metabolism in cancer, Estrogen signaling pathway, NOD-like receptor signaling pathway, Measles, Dorso-ventral axis formation, *Salmonella* infection	Viral carcinogenesis, Acute myeloid leukemia, Hepatitis C, AMPK signaling pathway, Drug metabolism - cytochrome P450, Pancreatic cancer, Legionellosis, Central carbon metabolism in cancer, Jak-STAT signaling pathway, Apoptosis

## Abbreviations

ATC: anaplastic thyroid carcinoma
PTC: papillary thyroid carcinoma
CDX: cell line-derived xenograft
FDR: false detection rate
